# Menstrual knowledge and practices of female adolescents in Vhembe district, Limpopo Province, South Africa

**DOI:** 10.4102/curationis.v38i1.1551

**Published:** 2015-11-26

**Authors:** Dorah U. Ramathuba

**Affiliations:** 1 Department of Advanced Nursing Science, University of Venda, South Africa

## Abstract

**Background:**

Although sexual issues are openly discussed in the media, sexuality and reproductive functions are treated as taboo. Menstruation is a normal physiologic process, but carries various meanings within cultures and is rarely discussed amongst families and communities.

**Purpose:**

This study sought to assess the knowledge and practices of secondary school girls towards menstruation in the Thulamela municipality of Limpopo Province, South Africa.

**Methods:**

A quantitative descriptive study design was used and respondents were selected by means of convenience sampling from a population of secondary school girls. The sample consisted of 273 secondary school girls doing Grades 10–12. A self-administered questionnaire was used to collect data, which was analysed by computing frequencies and percentages using the Statistical Package for Social Sciences (SPSS version 12).

**Findings:**

The findings revealed that respondents experienced menarche at 13 years and that menstruation is a monthly bleeding (80%) that happens to every female; it is a sign of adulthood (91%). 15% reported that it is the removal of dirt from the stomach and abdomen, 67% indicated the source of menstruation being the uterus, 65% the vagina and 13% from the abdomen. 73% reported having fear and anxiety at the first experience of bleeding and that they could not maintain adequate hygienic practices due to a lack of privacy and sanitary towels.

**Conclusion:**

Interventions are needed to increase girls’ opportunities to discuss menstruation and access information from adults including mothers, parents and guardians. School-based sexuality education should be comprehensive, begin early and be regularly repeated.

## Introduction

The menstrual cycle is the cycle of natural changes that occurs in the uterus and ovary as an essential part of making sexual reproduction possible. In human females, the menstrual cycle occurs repeatedly between the ages of menarche, when cycling begins, until menopause. Amongst girls, the first signs of puberty may emerge as early as 8 or 9 years and terminate at 15 or 16 years, with menarche. The average age of menarche of 12–13 years in most developing countries has been well established, with surveys showing that urban, educated, middle-class girls in many countries are now starting their periods on average at 12.5 years or earlier (World Health Organization [WHO] 2011). South Africa has also reported a similar mean age of menarche, and according to HIV & AIDS and STI National Strategic Plan (NSP 2007–2011), 6% of adolescent girls (15–24 years) reported having had sex by age 15 years. According to the SADHS (1998) and Ritcher and Mlambo (2005), Limpopo Province had the lowest contraceptive prevalence (54.9%), and poor contraceptive knowledge and accounts for the highest teenage pregnancy level in the country. The situation might be related to a lack of knowledge regarding the menstruation and how families and communities view menstruation, and the norms and values attached to menstruation. Studies that have been documented on sexual communication between parents and their adolescent children found that communication was minimal or not occurring at all (Ramathuba, Khoza & Netshikweta 2012). Menstruation is regarded as a normal physiological process; however it is viewed differently across cultures and families. Geronimus (2003) indicated that geographic, social or cultural variations influence teenage pregnancy and fertility; this can also imply that the meanings that families and communities attach to menstruation can differ. Arai (2007) indicated that the role of peers and other social factors in neighbourhood settings can be central to the idea of early childbearing communities. Adolescents, particularly in deprived areas where opportunities for social mobility are restricted, may be vulnerable to the influence of peers. Vha-Venda are a close knit community with less social movement and interaction and menstrual practices are contained in households by women elder and aunts (Vho-Makhadzi) and peers in social activities like khombani, domba, vusha and musevhetho. Adolescents can be influenced by their families, peers, neighbours and others in the community with respect to how menstruation is viewed, either as a natural transition, a cause for celebration, a passage to womanhood or a condition to be concealed because it is shameful (Patton & Viner 2007). The normal timing of first ‘menses’ amongst healthy girls extends from about 11 years to 14–15 years. Menstruations of 14 or 15 years are not unusual amongst rural girls or urban girls in low-income urban households (Awusabo-Asare *et al.* 2006; Salve *et al.* 2012; Singh 2006). The delay in menarche amongst rural girls and the low-socio-economic is attributed to the dietary practices. Knowledge of menstruation may be lacking amongst adolescents, as sex and sexual health carries a patriarchal gender-centred pattern, where opening a conversation on sexual reproductive health is uncomfortable or absent. In black rural communities information is conveyed by stipulated persons in the household, such as aunts, finding most mothers reluctant to provide information to their daughters. Adolescents usually get to be ill-informed by facts and myths surrounding menstruation from their peers. Dhingra, Kumar and Kour (2009) revealed:

that mothers, teachers, friends, relatives, television and books are the main source of providing information about menstruation to the adolescent girls. However, it is also seen that information received from these sources is often inaccurate and partial. (p. 44)

The way in which adolescent girls are prepared for their first menstruation may influence their reaction to it as well as the way they see themselves as young women. Amaral, Hardy and Hebling (2011) also argue that the more accurate the knowledge on menstruation at the time of menarche, the more likely will a woman’s menstrual experience be perceived as positive, which is associated with positive health habits and being more satisfied with her physical appearance. These experiences may exert an impact on feminine life and even on the way in which menstruation is valued. In most African communities preparation for menarche is lacking. Limited information is provided regarding physiologic issues at the expense of social and cultural factors. An adolescent experiences the new changed role of *khomba* – implying a young mature girl amongst black ethnic groups – by observing how adults treat their elder sisters and overhearing adult conversations regarding their views on menstruation. These result in adolescent girls having to internalise the myths and cultural stereotype of menstruation. Therefore, the study seeks to assess and describe menstrual knowledge and practices amongst female adolescents in Vhembe district, Limpopo Province.

## Problem statement

Menstruation and the menstrual cycle create a psychological discomfort for adolescents. Although menstruation is a natural process, it is often linked with several misconceptions and cultural practices. However, this depends upon knowledge and awareness of the subject which has an impact on the female adolescent in the event of menarche. Usually amongst black ethnic communities such discussions or information are not open, they are private and very discreet. Information given at this stage is generalised, and pertains to issues of how to behave and not ‘playing’ with boys without discussing the critical issues relating to menarche, conception, contraception, menstrual hygiene and reproductive health. Lack of menstrual information was observed in the same study, where adolescent girls could not specify the natural method of contraception as they could not relate the menstrual cycle with conception.

## Aim

The aim of the study was to assess and describe menstrual knowledge and practices amongst female adolescents in the Vhembe district of Limpopo Province.

## Objectives

Assess menstrual knowledge of female adolescents in the Vhembe district of Limpopo Province.

Describe menstrual practices of female adolescents in the Vhembe district of Limpopo Province.

## Research design and methods

A quantitative, explorative, descriptive design was used to assess menstrual knowledge and practices of secondary schools female adolescents at the Thulamela municipality, Limpopo Province. Explorative and descriptive studies were used to gain more information about menstrual knowledge and practices, as it provided a contextual picture of the situation. The target population included female adolescents aged 14–19 years in Grades 10–12 at six secondary schools. Non-probability convenience sampling was used to obtain a sample of 273 female adolescents, with 148 within the age range of 14–17 years and 98 being 18–19 years, whilst 27 had not specified their ages. The criteria for selection were all adolescent females who were already experiencing menstruation and were nulliparous. Systemic sampling of education circuits was conducted and six circuits were sampled and one secondary school randomly selected, which resulted to six secondary schools surveyed. Data was collected through self-report questionnaires that were distributed to female adolescents. Respondents in a survey answered the questions based on demography, knowledge of menstruation, attitudes, source of information and practices towards menstruation. Data was then manipulated by the Statistical Package of Social Science version 17, in order to describe the phenomenon (Polit & B.P. Beck 2008). Ethical approval was obtained from the provincial Department of Education, school principals and school governing bodies. The purpose of the study was given to the study respondents in their local languages. Female respondents were approached for their verbal and written consent. The confidentiality of the information was maintained by assigning identification numbers to each questionnaire. The respondents were informed that participation was voluntary, and that they could withdraw from the study at any time. Anonymity was maintained by ensuring that the names and identity would not be recorded. Participation in the study included informed consent from school governing bodies of the respective schools and written consent from the participants’ parents/guardians. A consent form was attached to the covering letter for parents/guardians to sign prior the distribution of questionnaires.

### Pilot study

A pilot study is a small-scale trail run on a limited sample from the same population as that of the final study before the main/final study is done (Polit & B.P. Beck 2008). A pilot study was carried out at Vhumbedzi circuit, where questionnaires were distributed to twenty female adolescents to determine the feasibility of the proposed study and to detect flaws in the questionnaire. No corrections emanated from the pilot study, and all questions were found to be clear.

### Validity and reliability

Validity refers to the degree to which the instrument measures what it is supposed to measure (Polit & C.T. Beck 2008). Content validity was achieved through a logical analysis of items, and was supported by literature review. Face validity involves the analysis of the questionnaire in view of face and content validity; to this end, the statistician endorsed the questionnaire for validity. Reliability was ensured by the test-retest method and internal consistency. The questionnaire was piloted to twenty female adolescents. The results of the pilot study were compared with those of the actual research. No discrepancies were observed, confirming the reliability of the instrument.

## Results

The results section attempted to obtain personal information about the respondents in order to contextualise the responses concerning sexuality issues against the knowledge of menstruation and practices by female adolescents.

### Socio-demographic information

Table 1 indicates that the majority of the respondents (53%) were aged between 15 and 17 years, with 36% between 18 and 19 years, whilst 10% fell within the ages that were unspecified (presumably, the unspecified years might been 20 years of age and above). Of the respondents 41% were in Grade 11, 35% were in Grade 12 and 24% of adolescents were in Grade 10. Nearly half of the respondents (46%) were living with both parents, followed by 41% staying with their mothers and siblings, whilst 6% were living with their fathers, 3% with grandparents, 1% with stepparents and 2% with an uncle or aunt. Hundred and sixty six (166) of the respondents’ mothers were unemployed, whilst 50 were professionals, 32 were labourers and 6 were clerks.

**TABLE 1:** Respondents’ age at menarche and knowledge of menstruation (*n* = 273).

Variables	Frequency	Percentage
**Age**		
9–10	2	2
11–12	33	12
13–14	196	73
Others	37	13
**Knowledge of menstruation**		
Yes	200	73
No	73	27

Table 1 demonstrates that 73% started menstruation at 13–14 years of age, 12% between 11 and 12 years, and only 2% menstruated at an earlier age. The findings suggest that the mean age for girls to start menstruating is 13 years. Two hundred (73%) of the respondents reported to have received information regarding menstruation, and only 27% had not received information regarding menstruation. The probability is that the information received was superficial on the basis that, if a girl matures, she gets a monthly period without understanding the context of menstruation in relation to conception and contraception.

### Menstrual knowledge

Regarding the knowledge of the participants prior to menarche, 27% reported knowledge of the physical changes that relate to menarche, 94% were aware of the social and religious restrictions, whilst (48%) were aware of hygienic practices and 98% were informed about the use of absorptive materials. Participants indicated that menstruation is a monthly bleeding (80%) that happens to every female; it is a sign of adulthood (91%). Fifteen percent reported that it is the removal of dirt from the stomach and abdomen. Regarding the source of menstruation, 67% indicated the uterus, 65% the vagina, whilst 11% indicated that it is from the abdomen, with 5% agreeing that it is from the stomach. Lack of sexuality information can have a detrimental effect on the sexual decisions in the reproductive life of the adolescent as it can affect sexual behaviour.

### Source of menstrual information

**FIGURE 1:**
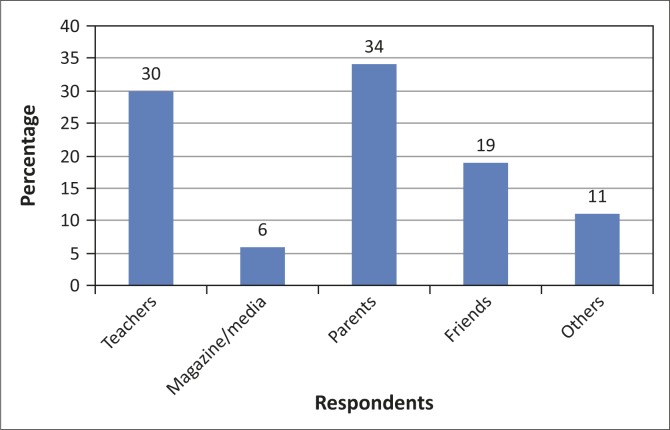
Respondents’ source of menstrual information.

Figure 1 indicates that 34% of the respondents had received information from parents, 30% from school, 19% from peers, 6% through magazines and 11% were from other sources like sisters or their love life. The majority of the respondents (34%) reported to have received this information at age 14, and most girls were already sexually active.

### Menstrual practices

Participants reported various practices related to menstruation: 37% reported to be using sanitary pads, 55% used cloths, 2% used newspaper and 26% used hand towels. Regarding the frequency of changing the absorptive material, 0.7% reported once, 95% twice and 7% thrice. Those who bathed once during menstruation were 58%, whilst 48% bathed twice. Method of disposing: 63% used the pit toilet; whilst 33% used refuse bins and 3% flushed the sanitary pads. Regarding caring for underwear, 7% washed and exposed these to sunlight, whilst 90% washed and hid the underwear, and 2% washed and discarded the underwear. When it came to the storage of underwear, 66% kept them clean and covered, whilst 29% kept them unclean and covered.

## Discussions

The age of the study participants ranged from 12 to 19 years. A similar study conducted by Ali and Rizvi (2010) reported the age of menstruating girls to range from 12 to 17 years. However, only 2 (0.7%) experienced their menarche earlier than that, which may be related to genetic and socio-economic factors regarding dietary intake. Table 1 indicates that 73% reported to have received information about menstruation, whilst 27% did not receive any information, but when further requested to indicate the physiologic changes associated with menarche, 27% had knowledge of the physical changes that relate to menarche, whilst 94% were aware of the social and religious restrictions that accompany menstruation, as culturally amongst Vha-Venda and Tsonga adolescents, they are restricted to be physically active and to contain themselves as young women (*vha songo tharamuwa- tharamuwa, vha di pute*). The majority of the respondents acknowledged that it is a monthly cycle and also sign of maturation. Maluleke and Troskie (2003) also indicated that during puberty rites, girls are taught the cultural rules of etiquette, obedience and are given sexuality education, suggesting that girls should not sleep with boys before marriage. Sexual health information relating to menarche forms the basis of women’s health. Figure 1 indicates 34% of the respondents reporting to have received information from parents, 30% from school, 19% from peers, 6% obtained their knowledge from magazines, whilst 11% were from other sources like sisters. The majority received information at home. Mothers usually play a significant role in imparting information, although the information is usually limited, as opening sexuality talk between mother and child can be an embarrassment. The results of this study are consistent with that of Amaral *et al.* (2011) that the preparation for menarche does not only provide girls with information concerning physiological issues, but involved emotional, cultural and social factors. Regarding the physiologic source of menstrual bleeding, 67% indicated the uterus, 65% the vagina, whilst 11% indicated that it is from the abdomen and 5% indicated the stomach as the source of bleeding. The findings are disturbing, as sexuality education is taught at schools during Life Orientation lessons in South African schools, which is a cause for concern, as culture inhibits discussions of such matters in public. Khanna, Goyal and Bhawsar (2005) concur with the findings and reported that 97.5% of the study participants in their study did not know the source of bleeding during menstruation. Adolescent girls tend to receive information about menstruation from a variety of sources, including parents, school, friends, and the media, and yet, despite the many sources of information, girls often report that the education they receive is insufficient in preparing them for menstruation. Sehar, Mohuddin and Ambreen (2012) reported that 12% of participants in their study were satisfied with the information received, whilst 84% still had questions in mind that needed to be resolved and nothing was explained regarding reproduction and only 2% were asked if they needed any clarification. These findings indicate that culturally it is not regarded important to give details on menstruation or queries that arise in the mind of the receiver, but to safeguard them against information that is believed to have an impact on the element of mutual respect which is maintained between mothers and their daughters. Menstrual knowledge is clouded by taboos and social cultural restrictions, resulting in adolescent girls remaining ignorant of the scientific facts and hygienic health practices necessary for maintaining positive reproductive health. Maluleke (2003) indicates:

that sexuality education should be available to all youth, at school and out of school, to address their sexuality health needs. They should have knowledge and an understanding of sexual development, human reproduction and healthy sexual behaviour. As puberty rites already address some aspects of sexual health, having more information on the sexuality education in the rites would strengthen the sexuality education given there and limit sexual problems that young girls experience. (p. 62)

Table 2 depicts that a minority of adolescents (37%) are reported to be using sanitary pads, whilst the majority (55%) use cloths, with the others using newspapers (2%) and hand towels. The situation is due to the socio-economic conditions of most teenagers, as their parents are unemployed and cannot afford sanitary pads; this makes most girls to be absent from school when they are experiencing their monthly cycle and could not change sanitary pads as frequently as needed. Furthermore, there are no proper disposal facilities at schools, because the pit toilets are available but dangerous, as most of them have fallen in and are in a dilapidated state. Most students relieve themselves outside and at backyard toilets, hence privacy is often jeopardised for those who might want to change their sanitary pads. This state of affairs contributes to students’ absence from school. Shah *et al.* (2013) also reported similar findings amongst Indian adolescents who used pieces of cloth which could absorb more menstrual blood, but they were still worried about spoiling their dresses, as the toilets in their schools did not have running water and in some cases toilets were non-existent at their workplaces (farms) and in most of their schools. Similar circumstances occur in Vhembe where adolescents stay away from school, for fear of spoiling their dresses. It was for these reasons that the radio station Phalaphala FM was prompted to start a campaign of collecting and donating sanitary pads to assist adolescent learners in need.

**TABLE 2:** Menstrual practices.

Variables	Frequency	Percentage
**Practices**		
Use of sanitary pads	100	37
Pieces of cloths	150	55
Newspapers	5	2
Hand towels	70	26
**How often do you change pads/cloth**		
Once	2	0.7
Twice	260	95
Thrice	10	7
**Frequency of bathing during menstrual period**		
Once	158	58
Twice	110	40
**Do you wash and discard**		
Yes	105	38
No	168	62
**Where do you discard**		
Pit toilet	173	63
Refuse bin	90	33
Flush in toilet	8	3
**Care of underwear/panty**		
Wash and expose to sun	20	7
Wash and hide	247	90
Hide and discard	5	2
**Storage of underwear**		
Clean and covered	190	66
Unclean and covered	80	29

Regarding caring for underwear, 7% washed and exposed these to sunlight, whilst 90% washed and hid and 2% hid and/or discarded the underwear. The hygienic practice related to menstruation is also affected by the socio-economic factors. Since most of the adolescents live with extended family members, they are forced not to bath, or when bathing, it has to be very early in the morning or late in the evening in order to maintain privacy. This arrangement results in the situation that they can therefore not wash their underwear and dry them appropriately. Shah *et al.* (2013) also cited similar findings related to bathing practices: due to lack of privacy, the girls took baths and washed their menstrual cloths early in the morning before other family members could wake up or invade their space. Sharing the room with other family members makes the adolescent girls to be discreet with their storage of underwear; thus they need to hide their underwear from other members because culturally it is frowned upon if they are left exposed. Unhygienic menstrual practices may affect their health and increase the risks of contracting diseases such as PIDs (Pelvic Inflammatory Diseases) and other complications. El-Lassy and Madian (2013) also suggest that during the menstrual period sanitary napkins should be changed about every four hours, possibly more often during the first days of the period when the menstrual flow is usually heavier. Changing menstrual pads before permitting leakage virtually prevents an unpleasant odour. Maree and Wright (2007) also reported the need for good perineal hygiene, as washing the genitalia infrequently during menstruation increases the risk for cervical cancer. Regarding the washing of underwear, most of the respondents washed and dried them not in direct sunlight and stored their underwear in plastic shopping bags, schoolbags and very few in a drawer. Thus ineffective washing and drying may lead to perineal infections such as thrushes. Those respondents who exposed their underwear to sunlight, hung them concealed by a washing cloth, thus obstructing exposure.

According to McPherson and Korfine (2004), girls are largely negatively directed about cultural beliefs concerning menstruation and the ways in which they will be expected to behave in order to uphold those beliefs. The perpetuation of cultural menstrual taboos that menstruation is ‘dirty’, that it must be hidden and should not be discussed in mixed company deprives adolescents the opportunity to get more information for them to take control of their reproductive health. Menstruation is usually associated with religious and cultural beliefs amongst black African cultures. Once you start menstruation, you are socialised not to sit and play with boys; and no sexual intimacy is permitted. In some instances one is not allowed to cook and wash the church clothes of her partner or husband for those attending Zion Christian Church; thus menstruation comes along with different practices across cultures. The findings are consistent with Santina *et al.* (2013), who reported that 15% of Indian adolescent girls of Gujjar did not participate in social ceremonies during menstruation. In addition, Indian adolescent girls did not participate in ceremonies (43.7%) or social activities with family (36.2%) during menstruation. In Saudi Arabia, young girls did not drink juice with vitamin C, indicating a change in the eating habits during menstruation. Such practices are detrimental to the reproductive health of adolescents, as they need to maintain health during menstruation like maintaining hydration and following a well-balanced diet to prevent secondary diseases that may occur, such as anemia.

### Limitations

The study was limited by the quantitative data collection method; if triangulated with focus group discussions, more in-depth information might have been sought.

### Recommendations

Mothers should be supportive to decrease their daughters’ negative reactions to menarche and to dispel their negative and secretive attitudes towards menstruation.

Maintaining good hygienic practices should be taught to maturing girls so that they understand the implications of their state of maturity.

Maturing girls should be empowered to view menstruation as a normal physiological process and not to shy away or keep it a secret for fear of embarrassment amongst peers if they matured early.

School health nurses should be made available to assist in enforcing sexuality education amongst female students with regard to menstruation, sex, teenage pregnancy, conception and contraception.

Involving premenarcheal girls in discussions about menstruation will help these girls to have a positive psychological and social mind-set about menstruation and dispelling the myths related to menstruation will assist young girls to positively embrace womanhood.

## Conclusion

Most adolescents lack scientific knowledge about menstruation and puberty. Parents often are reluctant to discuss this topic with their adolescent girls. Socio-cultural beliefs that accompany menarche and hygienic practices greatly impact negatively at the health of adolescents, and it is from this perspective that reproductive health education campaigns target puberty education so that young women may start adopting healthy lifestyles and develop adequate skills for sexual and reproductive health in future so as to achieve the millennium development goal of improving and reducing maternal mortality.
